# Guided by the principles of microbiome engineering: Accomplishments and perspectives for environmental use

**DOI:** 10.1002/mlf2.12043

**Published:** 2022-11-03

**Authors:** Haiyang Hu, Miaoxiao Wang, Yiqun Huang, Zhaoyong Xu, Ping Xu, Yong Nie, Hongzhi Tang

**Affiliations:** ^1^ State Key Laboratory of Microbial Metabolism, and School of Life Sciences & Biotechnology Shanghai Jiao Tong University Shanghai China; ^2^ Department of Environmental Systems Science ETH Zürich Zürich Switzerland; ^3^ Department of Environmental Microbiology ETH Zürich Eawag Switzerland; ^4^ College of Engineering Peking University Beijing China

**Keywords:** bioremediation, energy production, environmental microbiology, microbiome engineering

## Abstract

Although the accomplishments of microbiome engineering highlight its significance for the targeted manipulation of microbial communities, knowledge and technical gaps still limit the applications of microbiome engineering in biotechnology, especially for environmental use. Addressing the environmental challenges of refractory pollutants and fluctuating environmental conditions requires an adequate understanding of the theoretical achievements and practical applications of microbiome engineering. Here, we review recent cutting‐edge studies on microbiome engineering strategies and their classical applications in bioremediation. Moreover, a framework is summarized for combining both top‐down and bottom‐up approaches in microbiome engineering toward improved applications. A strategy to engineer microbiomes for environmental use, which avoids the build‐up of toxic intermediates that pose a risk to human health, is suggested. We anticipate that the highlighted framework and strategy will be beneficial for engineering microbiomes to address difficult environmental challenges such as degrading multiple refractory pollutants and sustain the performance of engineered microbiomes in situ with indigenous microorganisms under fluctuating conditions.

## 1 INTRODUCTION

Microorganisms live in communities and interact with their neighbors and environments. They drive global biogeochemical cycles, significantly change our living environments, and impact human health^1^, ^2^, ^3^. For example, approximately 60% of global wastewater is treated by microbial consortia involved in active sludge before being released into the natural water systems^4^, ^5^. Inside the human body, the number of microbial cells is 10 times higher than that of human cells, and human microbiomes have fundamental roles in physiology and health^6^. Moreover, breakthroughs are constantly appearing in the bio‐industry because of the capabilities of microbiomes to synthesize valuable products^7^, ^8^, degrade chemical pollutants^9^, ^10^, and produce biofuels^11^. Currently, a large number of important industrial chemicals and medicines have been produced by microbiomes, including isobutanol^7^, taxanes^8^, and hydrogen^11^. Although these achievements have affirmed the enormous potential of microbiomes for human use, formidable challenges remain in manipulating microbiomes for controllable output. Specifically, the ability to control the microbiome structure to sustain its function is still lacking. To address this challenge, a concept called “microbiome engineering” has emerged recently^12^, ^13^, ^14^. “Microbiome engineering” is a process to enhance the performance of microbiomes through targeted manipulation of the composition of natural communities (top‐down) or rational design and construction of new synthetic consortia (bottom‐up). Microbiome engineering is typically driven by general principles derived from a mechanistic understanding of the ecology and evolution of microbiomes. Such principles are presented as quantitative frameworks that can accurately predict the dynamics and function of a given microbiome and thus guide the rational engineering of microbiomes. This process of microbiome engineering provides a significant opportunity to further unlock the large potential of microbial communities.

Recently, an iterative “design‐build‐test‐learn” cycle (DBTL) was proposed as a general guideline for microbiome engineering^13^. Nevertheless, the development of microbiome engineering is still limited by knowledge and technical gaps. The major hurdle is the lack of quantitative theories and techniques to accurately measure, predict, and manipulate the structure and functions of microbiomes. Moreover, the interactions between many naturally occurring microbes are uncharacterized, and how these interactions are regulated by multiple environmental factors also remains poorly understood. Furthermore, tools to directly manipulate specific members of the microbial community have yet to be explored. In addition to these gaps, applying microbiomes to treat chemical pollutants in an open environment faces more challenges than applying microbiomes in a closed bioreactor. For example, degradation of multiple refractory pollutants (e.g., polycyclic aromatic hydrocarbons with high molecular weight, plastics, and halogenated compounds) in an open environment requires an engineered microbiome exhibiting diverse and high degrading abilities. In addition, fluctuating environmental conditions and indigenous microorganisms at contaminated sites affect the stability of engineered microbiomes. Inadequate tools for real‐time monitoring in the natural environment and policy restrictions on the use of genetically modified organisms (GMOs) limit the development of microbiome engineering for environmental use.

Integrating the theoretical research on microbiome engineering and its application achievements in recent years would advance the development of microbiome engineering in environmental science. Here, a set of microbiome‐engineering strategies is summarized from recent cutting‐edge studies. We then discuss how these strategies can be applied to advance the development of microbiomes for bioremediation. Based on present theories and practical accomplishments, we summarize and propose an approach that combines bottom‐up and top‐down approaches for the applications of microbiome engineering in natural environments. Finally, an “avoidance of toxic intermediates” strategy is discussed, which specifically addresses the environmental applications of microbiome engineering to avoid the build‐up of toxic intermediates that pose a risk to human health and to the environment.

## 2 PRINCIPLES AND STRATEGIES FOR MICROBIOME ENGINEERING

### 2.1 Common strategies for microbiome engineering

**Figure 1:**
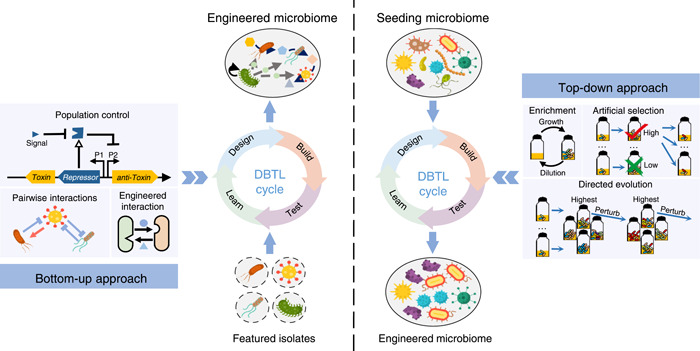
Two approaches to microbiome engineering. Left panel: the bottom‐up approach of microbiome engineering starts with several isolates, whose physiologic features have been well characterized. Coculture assays are performed to identify pairwise interactions among these isolates. Genetic engineering could be performed to design or modify the interactions. Systems based on cell‐to‐cell communications are used to directly control the behavior of specific populations. Right panel: the top‐down approach of microbiome engineering starts with a seeding microbiome containing uncultivated microorganisms. Then, microbiome engineering is managed to drive the seeding microbiome to self‐assemble into a stable system with highly optimized function. Three methods, including enrichment, artificial selection, and directed evolution, are commonly applied to perform top‐down engineering. No matter which approaches are used, the workflows should follow the “design‐build‐test‐learn” (DBTL) cycle proposed by Lawson et al.^13^.

Microbiome engineering aims to manipulate or de novo design a microbiome to achieve the desired functions^13^, ^15^, ^16^. Two approaches are commonly applied to design a microbiome: bottom‐up and top‐down approaches (Figure 1)^13^. The function of a multistrain microbiome arises from the individual members involved and from the interactions among these members^17^, ^18^, ^19^, ^20^. Thus, one could select or reconstruct suitable functional microorganisms as seeding members, identify or modify the interactions among these strains, and assemble a microbiome of these microorganisms, which is defined as the bottom‐up approach^18^, ^21^. However, it is still challenging to identify all the interactions within a multimember microbiome. For example, coculture assays of every two‐member combination are the common approach to identify pairwise microbial interactions; however, the number of pairwise combinations increases exponentially with the number of strains. Moreover, our understanding of how community‐level dynamics function in interaction networks is limited^22^, ^23^. Because of these complexities, microbiome engineering can alternatively follow a top‐down approach. This approach aims to select an efficient microbiome with the desired capacity from a natural community or a premixed community with natural strains with a well‐designed selection strategy and carefully optimize environmental variables^13^. These two approaches offer complementary strategies for engineering microbiomes with the desired functions.

To achieve the defined engineering goal, Lawson et al.^13^ adopted the practice guideline of the DBTL cycle from traditional engineering studies and suggested that such a cycle is also useful in engineering an effective and stable microbiome. The cycle starts with a rational design based on quantitative modeling informed by the given principles and assumptions, followed by the physical building of the microbiome. After testing the function and performance of the microbiome, researchers learn from failures and successes and thus incorporate new knowledge into subsequent cycles (Figure 1).

### 2.2 Microbiome engineering based on the bottom‐up approach

A bottom‐up design synthesizes a microbiome with defined naturally occurring strains or engineered strains. Microbial systems engineered using the bottom‐up approach are usually assembled using a limited number of members^24^, which are also termed “synthetic microbial consortia”^25^, ^26^, ^27^. Four principles have been proposed to guide the design of such consortia^12^: (i) control of intercellular interactions, (ii) control of spatiotemporal coordination, (iii) maintenance of robustness and stability, and (iv) prevention of biocontainment. This guideline has been widely used to engineer many simple but useful synthetic consortia^28^, ^29^, ^30^, ^31^. In addition to finding significant applications, studies based on these simple consortia have also developed numerous general principles for engineering microbiomes composed of many members.

#### 2.2.1 Principles from synthetic consortia of naturally occurring strains

The principles of how multiple ecological factors determine microbiome structure and function are fundamental to the rational engineering of microbiomes. These principles can be derived using synthetic consortia composed of naturally occurring strains (Table 1). For example, this approach has been used to explain the coexistence of competitive strains. One study predicted the survival of different strains in synthetic consortia based on the outcomes of every pairwise combination of strains^32^. The study also suggests that all strains that coexist in pairs will survive in a multistrain community; in contrast, strains that are excluded by any of the surviving strains will become extinct. A recent study further indicated that this principle could be extended to explain the evolution of microbial communities^56^.

**Table 1:** Summary of the principles of microbiome engineering derived from studies of synthetic consortia.

Consortium composition	Ecological factor	Proposed principle	Possible application	References
Natural strains	Pairwise coexistence	In a multispecies community, species that all coexist with each other in pairs will survive, whereas species that are excluded by any of the surviving species will become extinct	Maintain strain coexistence	[32]
Engineered strains	Growth rate	Controlling the growth of different members could maintain their coexistence and stabilize the community		[33, 34, 35]
Engineered strains	Cooperative interdependence	Cooperative interdependence contributes to the maintenance of the long‐term coexistence of multiple species		[36]
Natural strains	Temperature	Higher temperatures favor slower‐growing bacterial species in multispecies communities	Control the environmental factors	[37]
Natural strains	Nutrient availability	High nutrient concentrations often cause more negative than positive interactions between species, which exclude more species from the community, resulting in a loss of biodiversity		[38]
Natural strains	Nutrient complexity	The assembly of microbial communities in the presence of multiple nutrients is consistent with the behavior of consumer‐resource models		[39]
Natural strains	pH	Description of a set of interactions can be simplified using one simple environmental parameter, pH		[40]
Natural strains	Stochastic process	A rare successful colonist in the gut dominates the individual community and resists invasion by new colonists	Control the stochastic factors	[41, 42, 43]
Model derived	Pairwise interactions	Positive interactions enhance the diversity and productivity of the community but decrease its stability	Design interactions within the engineered microbiome	[44, 45, 46]
Engineered strains	Pairwise interactions	The dynamics of communities could be predicted using Lotka–Volterra pairwise Model parameterized by two‐strain coculture experiments	Develop quantitative modeling	[47, 48]
Engineered strains	Cooperative interdependence	Cooperative interdependence shapes intermixing the spatial pattern of the community, which helps to prevent cheaters from invasion	Design the spatial structure of the engineered microbiome	[49, 50]
Natural strains/Engineered strains	Cell–cell distance	Maintaining a suitable distance among the interacting strains could benefit community performance		[51, 52]
Engineered strains	Metabolic burden	Metabolic division of labor (MDOL) reduces the metabolic burden, giving higher metabolic efficiency	(1) Judge whether an MDOL strategy should be adopted. (2) Engineer a stable MDOL community with a defined structure	[8, 53]
Model derived	Metabolic burden; transfer efficiency	An MDOL community outperforms a single population only when the benefit derived from reduced metabolic burden overcomes the inefficiency of intermediate transport		[54]
Engineered strains	Parameters involved in a metabolic pathway	To maintain the stability of an MDOL community, the populations responsible for the initial steps in a linear metabolic pathway should hold a growth advantage (*m*) over the “private benefit” (*n*) of the population responsible for the last step. The steady‐state frequency of the last population is then determined by the quotient of *n* and *m*		[33]
Engineered strains	Substrate concentration and toxicity	The proportion of the population executing the first metabolic step in an MDOL community can be estimated by Monod‐like formulas governed by substrate concentration and toxicity		[55]

Synthetic consortia composed of naturally occurring strains have also been applied to test how important environmental factors, such as temperature^37^, nutrient availability^38^, nutrient complexity^39^, and pH^38^, ^40^, affect the compositions of multistrain consortia. For instance, higher temperatures are predicted to favor slow‐growing bacterial strains in multistrain communities^37^. Such synthetic consortia have also been used to study the potential impact of stochastic processes on community composition. Several studies have found that stochastic colonization of divergence between individual hosts is a key factor affecting the assembly of synthetic host‐associated microbiomes^41^, ^42^, ^43^. Accordingly, a “lottery”‐like assembly principle was proposed, suggesting that a rare successful colonist in the gut dominates the individual community and resists the invasion by new colonizers^43^.

One central question in microbiome engineering is how community‐level dynamics and functions arise from strain‐level interactions^22^, ^23^. Synthetic microbial consortia of strains with diverse interactions can be used to address this question. Using a bottom‐up approach, the dynamics of the synthetic gut microbiome^57^, ^58^, synthetic plant‐associated microbiomes^59^, ^60^, and synthetic food microbiomes^61^ could be accurately predicted by the outcomes of pairwise interactions. Several model analyses have provided theories linking such pairwise interactions to the community's structure, stability, and productivity^44^, ^45^, ^46^, ^62^. For example, positive interactions are predicted to enhance a community's diversity and productivity but decrease its stability^44^, ^45^. However, many of these predictions have not been experimentally tested because of inadequate tools for measuring the massive number of pairwise interactions in synthetic microbiomes composed of a large number of members. For example, a synthetic microbiome composed of 100 members would have 4950 pairwise interactions, which would require 155 96‐well plates to measure these interactions in triplicate for just one environmental condition^63^. A recently designed kChip device enables the parallel measurement of up to 10^5^ pairwise interactions^63^, ^64^, providing an opportunity to overcome this limitation. Accurate quantification of microbial interactions could improve our understanding of the ecology of microbiomes^65^, ^66^ and thus enhance our ability to engineer microbiomes with widespread applicability.

#### 2.2.2 Principles from synthetic consortia of engineered strains

For a natural community, the interactions between members may change with environmental conditions^67^, ^68^, and the underlying mechanisms are challenging to characterize fully. In this case, researchers can directly construct synthetic consortia with predefined interaction modes to avoid such unpredictable effects. This type of design can be achieved by engineering the metabolism of the member strains or mimicking cell–cell communications through the construction of genetic circuits, usually based on quorum‐sensing signals. Benefiting from this design, the interactions among members become simple and mechanistically clear. Therefore, controllable studies can be performed to determine how a given interaction mode affects community‐level properties. Moreover, these simple consortia also facilitate the construction of mechanistic models, thus enabling researchers to conclude general principles that can be used for other systems (Table 1)^12^, ^25^, ^69^.

One classic example is the construction of a consortium composed of engineered strains deficient in the synthesis of diffusible metabolites (such as amino acids and vitamins) and surviving by complementarily exchanging metabolites. This design was to create a cooperative consortium, CoSMO (cooperation that is synthetic and mutually obligatory), which is composed of two mutually beneficial *Saccharomyces cerevisiae* strains^36^. The authors found that cooperative exchange within the consortium could maintain long‐term community stability. Subsequent studies based on CoSMO further revealed that such cooperation drives the community to develop an intermixing spatial pattern^49^, which helps prevent invasion by cheaters^50^. Inspired by CoSMO, other similar consortia have been established using different bacterial strains^47^, ^51^, ^70^, ^71^, ^72^, ^73^. Several studies have proposed that the cost of metabolite production is a primary factor in determining the performance of a cooperative community^47^. A recent study revealed that two populations exchanged amino acids only within a short range (~5 μm)^51^. These studies offer many important principles for engineering a microbiome composed of more than two members. For example, introducing such interdependent interactions facilitates the coexistence of multiple strains that execute essential functions in the desired microbiome^74^, ^75^, ^76^, ^77^, ^78^. Furthermore, manipulating the spatial organization of a community^52^, ^79^, ^80^ to decrease the distance between interacting strains could increase their metabolic exchange^51^, potentially benefiting community performance. Similarly, engineering synthetic consortia containing other interaction modes provides other useful principles. Accordingly, the above‐mentioned principles summarized in Table 1 can be used to design and build stable and efficient microbiomes.

Synthetic microbial consortia can also be engineered to perform metabolic division of labor (MDOL). In such a consortium, the metabolic tasks involved in a pathway with many enzymatic steps are divided among different interacting strains^54^, ^81^, ^82^, ^83^. If a pathway is performed by a single microbial population, it requires that all of the necessary enzymes be produced by that population. Biosynthesis of these enzymes expends lots of energy and nutrients, thus creating a substantial metabolic burden that limits the growth of the population^54^, ^82^. In comparison, each member of an MDOL community only contains a subset of genetic components required for its respective metabolic step, resulting in a reduced metabolic burden^84^, ^85^. This perspective can explain the findings in several engineered communities engaged in MDOL, in which the metabolic efficiency of such communities was higher than that of the corresponding single‐population scenario^8^, ^53^. However, MDOL is not always a better strategy, and it has several limitations. First, the transport efficiency of intermediate metabolites could be limiting when the pathway segregates into different populations. Using mathematical modeling, a recent study found that a community performing MDOL outperforms a single population only when the benefit derived from reduced metabolic burden overcomes the inefficiency of intermediate metabolite transport.^54^ Second, the members involved in an MDOL community cannot stably co‐exist in some cases. In our recent study, we divided the degradation pathway of naphthalene into several steps and engineered several MDOL communities, in which each member was only able to perform one degradation step^33^. The MDOL communities are unstable because the strain performing the last step of the degradation pathway can obtain more benefits than other strains by privatizing available carbon sources. Using a mathematical model, we summarize several principles to predict the stability and assembly of MDOL systems based on simple pathway parameters (Table 1). A similar study suggests that the structure of the MDOL community can be rationally manipulated by changing the substrate concentration and toxicity^55^. In summary, these quantitative principles addressed two important questions in the engineering of a given pathway (Table 1): (1) whether engineering a synthetic consortium performing MDOL can increase the metabolic efficiency of the pathway and (2) how to engineer a stable MDOL community with a defined structure.

Different types of interactions in synthetic consortia have also been engineered using synthetic gene circuits^48^, ^86^, ^87^. For example, six two‐strain consortia executing six different social interaction modes can be engineered by the modular organization of well‐designed genetic modules^48^. Through this design, the study proved that the dynamics of communities composed of more members can be predicted from the behavior of a simple two‐strain community. Engineering cell‐to‐cell communication can also be performed to directly control the behavior of different populations involved in the microbiome^88^. In some cases, a faster‐growing population may lead to the collapse of the microbiome, so a programmed “self‐lysis” system can be introduced to control its population size, in which the cell self‐lyses upon receiving a quorum‐sensing signal^34^, ^35^. Such engineering approaches can even be used to generate oscillating dynamics in a synthetic consortium^89^, modulate the spatial patterning of a community^90^, ^91^, stabilize microbial strain ratios^92^, and coordinate the DNA cycling of different strains within a consortium^93^. These tools create opportunities to engineer microbiomes with designed compositions, spatial structures, and metabolic traits to achieve a defined engineering goal. A recent study developed an engineered consortium that enabled cell lysis in response to the concentration of an intermediate metabolite^94^. The intermediate released by the lysed cell could benefit the downstream metabolism mediated by the other population; therefore, the strategy improves the chemical production of the two‐strain consortium engaged in the division of labor (DOL).

### 2.3 Microbiome engineering based on the top‐down approach

Applications of the bottom‐up engineered microbiomes, however, are impeded by several limitations. First, many functionally important microorganisms remain uncultured. Second, establishing a mechanistic understanding of the interactions among the different microorganisms is challenging. Third, the practical application of engineered microbiomes for environmental use must ensure that the genetically engineered functions are not released into and do not disrupt natural ecosystems^12^, ^95^. As an alternative to the limitations of bottom‐up engineering, the top‐down approach could be used as it requires only a natural community, independent of the isolated strains, as well as a detailed understanding of microbial interactions. Moreover, as no GMOs are introduced into this system, it is more convenient and suitable to use in the natural environment.

**Figure 2:**
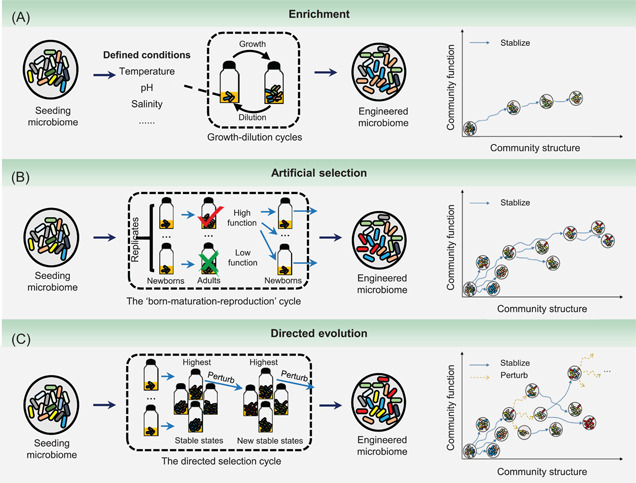
Three methods, enrichment, artificial selection, and directed evolution, are used for top‐down microbiome engineering. These methods are designed to drive the self‐assembly of a seeding microbiome into an engineered microbiome with the desired functions. (A) During enrichment, the seeding microbiome is introduced into the multiple growth‐dilution cycles under well‐defined environmental conditions. The microbiome is expected to gradually adapt to the environment and thus develop higher performance^101^. (B) In artificial selection, a collection of low‐density newborn communities is allowed to grow (maturation) into adult communities within a given time period. Then, the adult communities with enhanced functions are chosen to reproduce a novel generation of newborn communities to start the next cycle^103^. (C) One iteration of directed evolution starts with building a library of generationally stable communities with varied functions, and the community with the highest function is subjected to ecological perturbations^110^. As a result, a new library of generationally stable communities is generated to start a new iteration. As shown in the right panel of every graph, all these methods can be conceptualized as the shifts of a dynamic structure–function landscape, which represents the changes in the states with specific community structures and functions. Enrichment drives the evolution of the seeding microbiome along one trajectory into a final state with a generationally stable structure and an expected high function. The artificial selection first generates multiple states, and then, the states with higher functions are artificially chosen to continue the selection. As a result, a state with the highest function evolves from diverse evolving trajectories. In addition to the self‐assembled states, directed evolution also generates states by imposing ecological perturbations on the self‐assembled states, leading to more trajectories that benefit the selection of best‐performing communities.

Three common methods have been proposed to achieve top‐down engineering of microbiomes: enrichment, artificial selection, and directed evolution (Figure 2; Table 2). Enrichment is the most common method because it is the simplest strategy for manipulation. However, the defined conditions and simple manipulation may reduce the variability in community succession, which leads to potentially low functional diversity and redundancy that affects the function and stability of the engineered microbiome^101^. Alternatively, more diverse and efficient microbiomes are obtained when artificial selection of the microbiome is performed with more well‐designed selective cycles^108^, ^109^. Directed microbiome evolution is a recently proposed concept, inspired by the directed evolution of pure strains and biomolecules^110^. Regardless of the method applied, top‐down engineering has managed to drive the seeding microbiome to self‐assemble into a stable system with a highly optimized function, which is governed by the ecological and evolutionary principles involved^13^.

**Table 2:** Key strategies that increase the success of top‐down microbiome engineering.

Approach	Proposed way	Proposed strategy	References
Enrichment	Experiment	Optimize environmental variables	[96, 97, 98, 99]
	Experiment and model	Enrich microbiomes with multireplicates	[98, 100]
	Experiment and model	Nonspecific cross‐feeding supports species diversity	[98]
	Experiment	Control the size of the bottleneck	[101, 102]
Artificial selection	Model	Promote species coexistence	[103]
	Model	Suppress noncontributors	[103]
	Model	Choose additional communities besides the highest functioning ones to reproduce	[103]
	Model	Reduce stochastic fluctuations in the biomass of each member in “Newborn” communities	[103]
	Experiment	Control the incubation times between transfers: transfers need to be done at the peak of the selected phenotypic activity	[104]
	Model	Control the interactions that encourage species coexistence	[105]
	Experiment	“Breeding” several “Adult” communities to construct the “Newborns”	[106]
Directed evolution	Model	Methods to impose perturbations: bottleneck, species knock‐in, species knockout, migration from the pool, coalescence, and altering resource concentration	[107]

#### 2.3.1 Top‐down microbiome engineering via enrichment

Enrichment of a microbiome is performed by imposing a seeding microbiome into multiple dilution‐growth cycles under well‐defined environmental conditions (Figure 2A)^101^. The outcome of enrichment is primarily affected by environmental variables, such as temperature^96^, nutrient availability, pH^96^, ^97^, energy source^111^, ^112^, and carbon source^96^, ^98^. Enriching microbiomes with multiple replicates has recently been considered a powerful approach to developing a quantitative theory of microbial ecology^100^. For example, Goldford et al.^98^ found that the composition of the enriched microbiomes under steady‐state conditions converged based on experimentally imposed conditions rather than the strain composition of the initial inoculations. These results are highly predictable when using classical ecological models that consider the main factors of nutrient availability and nonspecific cross‐feeding. Another study found similar results, showing that the composition of microcosms using the same carbon source (petroleum hydrocarbons or asphaltene) was highly similar and had core microbiomes mainly consisting of petroleum‐degrading bacteria^99^. Therefore, optimizing environmental variables may be more efficient than screening diverse initial seeding microbiomes to establish high‐performance enrichment microbiomes.

The enrichment process is also driven by stochastic factors. For example, the bottleneck effect in a community refers to a sharp reduction in community size owing to environmental events^41^, ^113^, ^114^. Such a stochastic effect can cause the loss of strain diversity, thus affecting community function. Dilution manipulation in each cycle reduces the community size and thus imposes a bottleneck^102^, ^115^, ^116^, ^117^. Studies have suggested that decreasing the size of the bottleneck (reflected by the increasing dilution factor) is more likely to remove rare taxa^118^, ^119^ that may possess key functions and significantly influence microbial diversity^120^. Nevertheless, one study also showed that the increasing dilution factor is not proportional to the corresponding reduction in the diversity and functionality of the community^121^. Therefore, determining an optimal dilution factor is a critical step to obtaining a good top‐down engineered microbiome through the enrichment approach.

#### 2.3.2 Top‐down microbiome engineering via artificial selection

Recently, microbiome selection using a “born‐maturation‐reproduction” cycle has been proposed^103^ (Figure 2B). Each selection cycle starts with a collection of low‐density “Newborn” communities, which grow (maturation) into “Adult” communities within a given time span. Then, several specific adult communities are chosen to “reproduce” such that each is randomly partitioned into multiple newborn communities to start the next cycle. Over sufficient selection cycles, microbiomes with the desired functional traits were obtained. This framework suggests that natural microbial communities can be selectively bred to produce engineered microbiomes with desirable features, as has been done with animals and plants for thousands of years^122^. Compared with the enrichment approach, artificial selection usually starts with more replicates. Importantly, at the end of one cycle, only a specific number of adult microbiomes (normally those with top performance) were selected for seeding in the next cycle (Figure 2A,B). With this approach, microbiomes are continually selected to perform better in the desired function during the selection cycles, resulting in better outcomes than those obtained using the enrichment approach.

Many efforts have been made to develop general principles to guide the artificial selection of microbiomes. A standard protocol for the artificial selection of microbiomes was developed and used to establish a root‐associated bacterial microbiome that confers salt tolerance to the plant^123^. Four essential aspects of artificial selection were proposed by simulating the selection process in silicon: (1) promoting species coexistence, (2) suppressing noncontributors, (3) choosing additional communities besides the highest‐functioning ones to reproduce, and (4) reducing stochastic fluctuations in the biomass of each member strain in newborn communities^103^. These model predictions were partially verified through experiments in another study, which used a defined set of strains or soil communities as the starting “Newborn”^124^. Another experimental study demonstrated that optimizing incubation times is crucial for preventing the loss of strains with key functional traits during artificial selection^104^. In a recent study, individual‐based modeling (IBM) showed that interactions that encourage strain coexistence restrict the compositions of newborn communities, which drives the microbiome away from maximal function and decreases its heritability^105^. Newborn communities in artificial selection can also be built by “breeding” different adult communities. Another study found that “breeding” several adult communities to construct newborns was more efficient in biomass production than using a single adult community^106^. Overall, the strategies developed from these studies could be adopted for further top‐down engineering of microbiomes.

Although artificial selection is considered a useful top‐down engineering strategy, it has two main limitations^110^. First, artificial selection of microbiomes requires that the adult communities after each cycle exhibit considerable variation in the selected function; therefore, adult communities with higher functional performance can be selected for breeding. However, the variation usually decreases rapidly over cycles because the selection process continually eliminates strains from the metacommunity, after which all adult communities are dominated by the same selectively favorable strains. Without mechanisms that regenerate between‐community variation, selection cannot continue after several cycles. Therefore, approaches benefiting from the regeneration of this variation are critical to continue artificial selection after the first few rounds. Second, the newborn communities may not exhibit the dynamics and functions of their adult communities. This generational instability may invalidate top‐down engineering.

#### 2.3.3 Top‐down microbiome engineering via directed evolution

To overcome the limitations of artificial selection, Sánchez et al. proposed that directed evolution could be a new tool for top‐down engineering of microbiomes^110^ (Figure 2C). This concept first assumes that adult communities can converge into several structurally and generationally stable communities within several selection cycles and that communities with the same structure show the same functional traits. Accordingly, one iteration of directed evolution starts by building a library of generationally stable communities with varied functions through multiple‐replicated artificial selection. Then, the community with the best function is chosen and exposed to perturbations using different methods, resulting in a new library of generationally stable communities to start a new iteration. Theoretically, this process can last longer than normal artificial selection because functional variation among adult communities within a library always exists. A key step in directed evolution is the imposition of perturbations. Possible approaches include methods used in enrichment and artificial selection (bottleneck, propagule breeding) and other methods, including species knock‐in, species knockout, and altering resource concentration. Thus, it combines the components of enrichment and artificial selection to form a more rational framework. This concept has been tested in silico, suggesting that directed evolution using several perturbation approaches is more effective for top‐down engineering^107^. The study also found that directed evolution could produce communities that are more stable against ecological perturbations. However, the conceptual appeal has not been tested experimentally.

### 2.4 Mathematical models in microbiome engineering

Mathematical modeling is a powerful tool for establishing a quantitative understanding of how different factors affect the functional dynamics of microbiomes (Table 3). This understanding offers theoretical guidance for the rational engineering of microbiomes^13^, ^132^, ^133^, ^134^. A mathematical model is usually built on simple assumptions that define the basic features of a focal microbial system. The mathematical model uses experimentally measured parameters as inputs to determine whether the experimentally measured dynamics or functions of the microbiome can be predicted by the given assumptions. If it shows strong predictive power, one could develop a simple framework linking the limited set of assumptions with community properties. Thus, it plays an important role in “learning” knowledge after the “test” stage. The new theoretical knowledge can be then used as a guide to “design” a novel microbiome in the next DBTL cycle^13^.

**Table 3:** Mathematical models used for microbiome engineering.

Approach	Model	Main model input	Feature	References
Bottom‐up engineering	Lotka–Volterra pairwise model (LVPM)	Pairwise interactions	Based on pairwise interactions	[32, 47]
	Lotka‐Volterra mechanistic model (LVMM)	Interaction mechanisms	The model structure may be more complex than LVPM but can be simplified or solved by simulations	[33, 54, 89]
	Genome‐scale metabolic model (GSMM)	Genomic data	Link the metabolic traits of a single member with the properties of the community	[31, 61, 125, 126]
	Individual‐based model (IBM)	Interaction mechanisms; spatial position	Simulate the dynamics of a community in spatially structured environments	[76, 127, 128, 129]
	Computaion of microbial ecosystems (COMETS)	Genomic data; spatial position	Combine features of IBM and GSMM	[130, 131]
Top‐down engineering	Consumer‐resource model (CRM) integrated with ecological interactions	Parameters regarding nutrient availability, interactions, etc.	Predict the dynamics of the enriched multispecies community	[98, 107]
	IBM	Parameters regarding nutrient availability, interactions, etc.	Capture the dynamics of the multispecies community with artificial selection	[105]

The models used for bottom‐up engineering of microbiomes are diverse. With the simplest assumptions, the Lotka–Volterra model uses only a few parameters to quantify the growth of each population and the interactions between these populations. Conversely, pairwise interactions measured by experiments can be incorporated into the Lotka–Volterra model to generate the Lotka–Volterra pairwise model (LVPM). LVPM was applied to describe the interactions between different amino acid‐deficient strains in engineered synthetic consortia and accurately predict the experimental outcomes^47^. Variants of LVPM are useful for establishing simple assembly principles for microbial communities^32^. In addition, the Lotka–Volterra model can also be combined with the molecular mechanisms of the interactions, generating the Lotka–Volterra mechanistic model (LVMM). A previous study proposed that the presence of diverse metabolic interactions makes the LVPM prediction unreliable^135^, and LVMM should be used in such cases. However, the introduction of multiple mechanisms increases the difficulty of solving these mathematical systems. This system can be simplified by imposing reasonable assumptions. For example, principles governing the function^54^ and assembly^33^ of microbial communities engaged in MDOL can be derived from the simplified LVMM model.

The genome‐scale metabolic model (GSMM) is another useful model for predicting community properties. It could predict the microbe–microbe and microbe–environment interactions based on the genetic information of a pure strain, and thus, it is ideal for bridging the gap between the metabolic traits of a single member with the properties of the community^33^, ^125^, ^136^. Recent advances in large‐scale computation and genome sequencing have enabled the description of community dynamics by integrating 1500 GSMMs established from the genomes of human‐associated bacteria^137^. Furthermore, the IBM is typically used to simulate the dynamics of a community in spatially structured environments with discrete conditions^138^. IBM is built based on the characterization of the individuals involved in a community. Therefore, it can be used to examine how single‐cell traits such as cell positioning^127^, cell morphology^128^, and contact‐dependent interaction^129^, impact community‐level properties. Incorporated with the assumptions regarding evolutionary events (e.g., mutation and the evolution of new genotypes), it could also simulate the evolution of microbiomes in spatially structured environments^76^. To simulate bottom‐up established microbial ecosystems, Dukovski et al. combined the GSMM and IBM and developed a metabolic modeling platform named computaion of microbial ecosystems (COMETS)^130^. Benefiting from the merits of both models, COMETS can predict the spatiotemporal dynamics of microbial ecosystems that result from the intracellular metabolism of individual strains.

A limited number of models have been applied to simulate top‐down engineering cycles. The consumer‐resource model is a classical ecological model that successfully captures the dynamics of resource competition among different strains. Dynamics derived from the consumer‐resource model primarily support the “competitive exclusion principle,” which suggests that two strains competing for the same growth‐limiting resource cannot coexist^139^. However, this prediction does not completely match the case of top‐down engineered microbiomes. By integrating ecological interactions, such as metabolic cross‐feeding, this limitation was overcome and the novel model was used to accurately capture the coexistence of different strains involved in the enriched microbiomes^98^. This model was further applied to construct a quantitative framework for directed evolution^107^. Alternatively, IBM has been used to search for artificial selection strategies^123^.

## 3 ACCOMPLISHMENTS AND PERSPECTIVES FOR ENVIRONMENTAL USE

Researchers have adopted the concept of “microbiome engineering” to overcome the challenges of bioremediation, including degradation of refractory pollutants (polycyclic aromatic hydrocarbons with a high molecular weight^140^, plastics^141^, halogenated compounds^142^, etc.), and emerging pollutants such as pharmaceuticals and personal care products^143^. In addition, it has been applied to deal with contaminated sites with high concentrations of multiple contaminants (heavily polluted soil, landfill leachate, industrial wastewater, etc.). Here, the achievements in microbiome engineering for environmental use via bottom‐up and top‐down approaches are reviewed. We then further summarize perspectives on how to apply the recently proposed theories, conceptualize a combined framework, and propose a specific strategy for environmental use.

### 3.1 Bottom‐up microbiome engineering for environmental use

Bottom‐up microbiome engineering (i.e., synthetic microbiome) provides ecological principles and mechanistic insights into building microbial consortia to enhance pollutant removal. First, synthetic consortia engaging in the DOL were constructed to degrade pollutants with complex structures^30^, ^144^, ^145^, ^146^. Several studies suggest that compared with the single strain containing the complete metabolic pathway, the DOL engineering could significantly reduce the metabolic burden (the energy cost of one population performing its specific functional step) for each member of the synthetic consortia^145^, resulting in higher degradation efficiencies. Notably, although DOL potentially reduces the metabolic burden, increased demands for intermediate transport and nutrient competition among the members may also reduce the degradation efficiencies. These limitations are minimally considered in the above‐mentioned studies adopting the DOL strategy (Table 1). Second, the degrading capability of synthetic consortia can be further enhanced by reducing the growth‐inhibiting intermediates, such as H_2_S produced in aniline biodegradation^145^ and nitrophenol produced in parathion biodegradation^10^. A general approach is to introduce new members capable of degrading harmful intermediates into the consortia to relieve the accumulation of intermediates^145^. Third, the synthetic consortia, consisting of members with different metabolic functions, can degrade multiple pollutants simultaneously, which can also substantially reduce the metabolic burden compared to the strain possessing all abilities. This strategy was used to construct synthetic consortia for degrading multicomponent pollutants, such as BTEX (benzene, toluene, ethyl benzene, and xylene isomers)^146^ and alkanes^147^. Thus, it is thought to be important in the remediation of multiple contaminants.

Despite these significant achievements, many challenges remain in engineering microbiomes by the bottom‐up approach for environmental use. As mentioned above, DOL may result in increased demands for intermediate transport and nutrient competition among the members. In this case, researchers can apply the recently proposed mathematical frameworks (e.g., the equation derived by Tsoi and colleagues) to integrate specific pathway parameters. This kind of framework can determine whether the DOL engineering is able to increase the efficiency of the pathway of interest before performing microbiome engineering^54^. Moreover, the engineering of a DOL consortium is quite limited by the poor understanding of metabolic pathways or the networks of interactions involved in degrading the targeted pollutants, especially refractory compounds. To bridge this gap, many studies first perform meta‐omics and metabolic model investigations to establish a basic understanding of the degradation pathways and microbial interactions^148^, ^149^, ^150^, ^151^. Collecting prior knowledge using omics‐based methods can guide the rational design and optimization of synthetic consortia toward higher degradation efficiency and stability^151^.

Another common issue faced in bottom‐up engineered communities is that the strains involved cannot maintain stable coexistence because of the competition for common resources and space. We summarize three possible strategies from recent studies that could solve this issue (Table 1). First, pairwise interactions among every involved strain should be measured, and the investigators must ensure that all members coexist with each other in pairs to survive. According to the principle proposed by Friedman et al.,^32^ coexistence can be achieved among multiple members involved in the consortium. Second, strains can be introduced to achieve interdependent interactions. For example, naturally auxotrophic strains can be rationally grouped to achieve such interdependence. However, the construction of consortia using naturally occurring strains is empirical and may not be well‐tuned, as it is difficult to manipulate the interactions between strains. Alternatively, synthetic consortia can be constructed using GMOs with defined functions. Genetic modules for population control can be imposed on fast‐growing strains to balance the overall growth and allow slow‐growing strains to perform the desired functions. The risk of the release of GMOs in other ecosystems can be prevented by developing more efficient and stable “suicide strategies”^152^, ^153^, ^154^. But currently, the construction of synthetic consortia with engineered strains for environmental use is much less common than using naturally occurring strains, due to the policy restriction for using GMOs in open systems and the lack of GMOs with truly desirable functions. Finally, when a consortium is initially constructed, it is important to regulate the structure of a consortium to achieve better efficiency, which can be achieved by modulating abiotic factors (e.g., temperature, nutrient availability and complexity, and pH; Table 1) or biotic factors (e.g., changing the relative growth rate of strains^33^).

### 3.2 Top‐down microbiome engineering for environmental use

Compared with the bottom‐up approach, the enrichment‐derived top‐down approach is independent of metabolic mechanism understandings and does not involve GMOs. It has been applied widely in bioindustrial applications, including activated sludge^155^, granular sludge^156^, and compost^157^. Several conceptual ecological models of activated and granular sludge in wastewater treatment have been developed^156^, ^158^. These models provide important guidelines for the further optimization of performance via changing environmental factors, such as temperature^159^ and organic composition^160^, ^161^. In addition, novel models that not only incorporate traditional modeling approaches based on the fundamental information of “core microorganisms” but also include microbial metabolic networks and substrate preferences of different members under alternating anoxic–oxic conditions allow researchers to rationally preset the primary microbial composition^162^. This should provide for a more rational and predictable development of routine bioremediation technique.

Many pollutant‐degrading microbiomes have also been enriched in the laboratory by systematic control of culture conditions^163^, ^164^, ^165^. These studies offer useful microbiomes, strains, and genes for further mechanistic insights into degrading different pollutants (petroleum hydrocarbons, phenanthrene, lignin, etc.). Notably, the dilution‐to‐stimulation and dilution‐to‐extinction approaches were combined to enrich a minimal and effective lignocellulolytic microbial consortium^166^. In this study, the initial lignocellulolytic consortium was enriched from the inoculum source by stimulating the plant‐residue‐degrading microbes using a mixture of three different plant residues and dilution‐to‐stimulation cycles. A minimal and effective consortium consisting of only two species was then achieved through continued serial dilution to extinction from the initial lignocellulolytic consortium. Such a combined top‐down strategy not only guides the assembly of effective microbial consortia but also provides a simple inoculum source to isolate functional strains for the more rational construction of synthetic consortia using a bottom‐up approach.

However, the enrichment with one given setup (i.e., a given set of environmental factors and one constant dilution factor) usually leads to a deterministic outcome, which may not possess optimal efficiency. In contrast, artificial selection and directed evolution introduce variations to each dilution‐growth cycle, creating opportunities to continuously select for better degradation efficiency. For example, an efficient 3‐chloroaniline‐degrading consortium was obtained via artificial selection. In this case, after 4 days of incubation, three tubes with the highest 3‐chloroaniline degrading ability were mixed as the seed of a “new generation.” After 30 such selection cycles, the 3‐chloroaniline‐degrading ability of later generations significantly improved^167^. In this way, engineered microbiomes can be continuously obtained with better pollutant degradation efficiencies for environmental use^104^. However, the strategies derived from the recent understanding of artificial selection and directed evolution (Table 2) have not been applied to the top‐down engineering of microbiomes, although they are expected to increase the success rate. Thus, we encourage the application of artificial selection and directed evolution in future endeavors.

In addition to enrichment‐dependent top‐down approaches, a recent in situ microbiome engineering method provided an alternative strategy to manipulate the composition and function of a natural or top‐down engineered microbial community. Combining environmental transformation sequencing (ET‐seq) and a DNA‐editing all‐in‐one RNA‐guided CRISPR‐Cas transposase (DART) system, it enabled gene‐ and species‐specific editing in a community context^168^. Based on meta‐omics data, researchers can use the ecological network of the consortium to identify its driver species (core species) and their competitors^169^. Among them, species that can be edited in situ will be screened out through ET‐seq. The relative abundance of the driver species can be improved by either enhancing their own capability or inhibiting the growth of their competitors. Alternatively, with mechanistic insights into the metabolic network of the driver species, efficiencies of the consortium can also be improved by enhancing the degrading pathways or removing the unexpected byproduct pathway. Compared with the enrichment‐dependent approach, the in situ edited consortium is easier to predict and more diverse. In addition, because the in situ edited member is one of the indigenous organisms, it will be more compatible with other indigenous organisms. Although the in situ microbiome editing approach has only been performed in model communities, it may enhance the ability to engineer microbiomes for environmental use.

### 3.3 Framework for combining top‐down and bottom‐up approaches

Previous studies have mostly used top‐down and bottom‐up approaches separately. We suggest that the two approaches are strategically complementary. First, the top‐down approach can directly obtain microbiomes with a high degrading capability without a prior understanding of degradation mechanisms (whereas the bottom‐up approach requires such understanding). Second, the bottom‐up approach aims to construct a microbiome de novo with clear mechanisms so that it can be manipulated more easily than the top‐down microbiomes. Therefore, a rational integration of both approaches can leverage their strengths and mitigate their weaknesses. Several studies show the utility of combining both approaches^165^, ^170^ such as the plant microbiome bioremediation system for petroleum hydrocarbons^148^, ^171^ and the atrazine case^165^.

**Figure 3:**
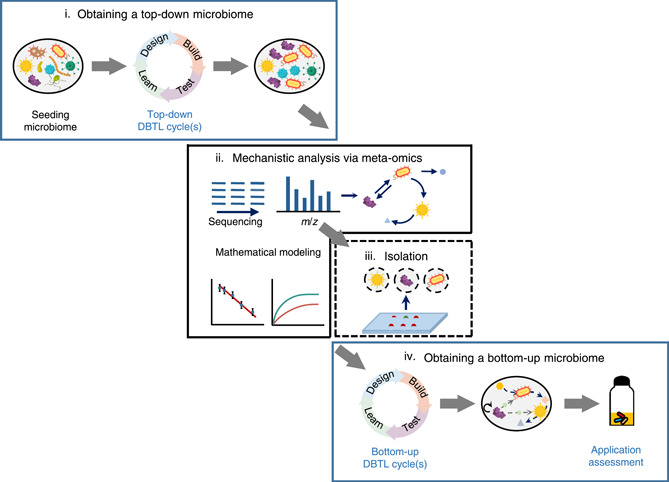
Schematic diagram for the combined framework of top‐down and bottom‐up approaches for environmental use. (i) Preliminary microbial community with a degrading capability of interest is derived from a seeding microbiome by one or several top‐down DBTL cycle(s), in which enrichment, artificial selection, or directed evolution could be applied. (ii) Mechanistic insights of this top‐down engineered microbiome can be identified by meta‐omics (metagenomics, metatranscriptomics, metaproteomics, or metabolomics), which is incorporated into mathematical modeling to predict interactions among the members. (iii) Based on the mechanistic insights, microorganisms of interest can be isolated by enrichment or high‐throughput single‐cell sorting and then used for further bottom‐up microbiome engineering. (iv) Bottom‐up design‐build‐test‐learn (DBTL) cycle(s) would be conducted, guided by mechanistic insights, to engineer a new bottom‐up microbiome with higher degrading efficiency and assessed in its effectiveness in the intended application.

Here, we systematically summarize these interesting achievements and propose a possible framework that combines one DBTL cycle of the top‐down approach with another cycle of the bottom‐up approach, as shown in Figure 3. First, obtain a top‐down microbiome through one or several DBTL cycle(s) to engineer a microbial community with the appropriate degrading capability. Second, develop mechanistic insights into the top‐down microbiome by elucidating why the community works efficiently and how it can be improved with a rational bottom‐up approach. For example, important metagenome‐assembled genomes (MAGs) could be extracted through metagenomic analysis of the top‐down community and then GSMMs can be built based on these MAGs to better understand the principles governing the dynamics and functions of the community. Third, isolate microorganisms with desirable functions. Guided by meta‐omics data, a “core list of microorganisms” can be developed and used to isolate microorganisms with the desired functions. The specific microorganisms can be enriched or isolated using specific media or culture conditions. As an alternative, a high‐throughput single‐cell sorting technique could also be used. Fourth, construct a synthetic consortium using a bottom‐up approach. The bottom‐up DBTL cycle(s) could be further modified by adding beneficial strains with defined functions or reconstructing the consortium de novo using the bottom‐up approach.

### 3.4 Proposed strategy specific to environmental use

The biodegradation usually results in the detoxification of pollutants. However, such biotransformation may also produce intermediates that are more harmful to humans than their parent compounds, especially in the case of refractory pollutant transformations^172^, ^173^. For example, 1,4‐dicarboxybenzene (BDC) is a major intermediate in the degradation of butyleneadipate‐co‐terephthalate. While butyleneadipate‐co‐terephthalate itself is harmless to humans, the accumulation of BDC can impact human health and the soil environment^172^. In addition to the intermediates with known toxicological effects (e.g., BDC), the toxicities of many intermediates during refractory‐pollutant degradations have not been clearly evaluated. Some of them can be easily detected in the metabolism of the parent compound but we know very little about how they are further degraded (e.g., 2‐hydroxy‐4‐(3′‐oxo‐3′H‐benzofuran‐2′‐yliden)but‐2‐enoic acid in the dibenzofuran‐degrading pathway)^174^, ^175^.

Thus, the potential damage of toxic intermediates should be taken into consideration during the consortium design. Here, we propose a specific strategy of microbiome engineering for environmental use: “avoidance of toxic intermediates,” which aims to avoid the build‐up of toxic intermediates that risk human and environmental health. The intermediates that naturally accumulate in initial microbiomes should be determined. Critical intermediates in regulating the microbial physiologic activities and interactions (e.g., the effectors for quorum sensing or transcriptional regulation) will then be distinguished from those that can harm the human body. The accumulation of the latter type needs to be prevented. With a clear understanding of degradation pathways, the accumulation of toxic metabolites can be prevented by knocking out the pathways that generate them or overexpressing genes involved in degrading them. These genetic manipulations can be performed in the member(s) of a bottom‐up engineered microbiome or in indigenous microbes from a top‐down engineered microbiome through in situ engineering. If the knowledge of intermediate degradation pathways is unclear, naturally occurring strains with the degradation function need to be introduced and enriched in the engineered microbiome. Alternatively, we should set the “prevention of chemical contaminants” as another goal in addition to the degradation efficiency of the substrate when performing the top‐down approach of a microbiome.

## 4 CONCLUDING REMARKS

In summary, researchers can rationally design an efficient, stable, predictable, and safe microbiome through microbiome engineering. With present mathematical models, high‐throughput culturing techniques, and quantitative analysis tools, the accuracy of predicting the structure and function of a microbial community has recently been improved. In the future, we should investigate how to rationally regulate microbiomes or de novo construct microbiomes to achieve desired functions through environmental factors, microbial interactions, and physical characteristics of involved strains. To this end, we need to test the feasibility of recently proposed principles of microbiome engineering under more complex conditions (e.g., in situ environments) and integrate different useful strategies. Benefiting from these new tests, novel quantitative principles can be proposed to increase our understanding of microbial ecology and guide future microbiome engineering. We expect that the combined framework of top‐down and bottom‐up approaches can be applied to address the enormous environmental challenges for bioremediation (Table 4).

**Table 4:** Accomplishments and perspectives of microbiome engineering strategies.

Approach	Strategy	What we have done	What we can do
Bottom‐up engineering	With naturally occurring strains	Successfully applied for construction of the microbiome to degrade specific compounds in the lab and practical bioremediation	Maintaining long‐term stability and coexistence of the members
	With engineered strains	Synthetic consortia were constructed by the DOL approach, such as dividing the pollutant mineralization pathway into different strains	Maintaining long‐term stability and enhancing metabolite‐exchange efficiency. Mathematical models proposed by Lingchong You et al. can help guide the engineering of metabolic pathways by the DOL approach^54^. Developing population control techniques for GMO restriction
Top‐down engineering	Enrichment	Successfully applied for construction of microbiomes to degrade specific compounds in the lab and practical bioremediation	Optimization of imposed perturbations, including various environmental conditions, and dilution factors guiding the top‐down engineering process with the ecological models and mathematical models, such as bottleneck, resource shift, and species knock‐in^107^. If possible, using artificial selection and directed evolution before enrichment
	Artificial selection	After dozens of selection cycles, the late generations are more efficient than the seeding microbiome	
	Directed evolution	Only in theory	
